# Sense of coherence in bipolar disorder– a longitudinal study

**DOI:** 10.1186/s12888-025-06779-3

**Published:** 2025-04-02

**Authors:** Erik Pålsson, Mikael Landén

**Affiliations:** 1 Institute of Neuroscience and Physiology, Sahlgrenska Academy, Gothenburg University, Gothenburg, Sweden; 2 Department of Medical Epidemiology and Biostatistics, Karolinska Institutet, Stockholm, Sweden

**Keywords:** Bipolar disorder, Sense of coherence, Longitudinal study

## Abstract

**Background:**

The Sense of Coherence (SOC) scale was designed to measure an individual’s ability to perceive life as comprehensible, manageable, and meaningful. While low SOC is associated with various psychiatric disorders, its association with bipolar disorder remains unclear. This study explores SOC in individuals with bipolar disorder, its associations with clinical characteristics, and its stability and predictive value over 14 years.

**Methods:**

We included 248 individuals with bipolar disorder and 113 healthy controls from the St. Göran Bipolar Project. SOC was assessed using Antonovsky’s 29-item scale at baseline and after 14 years. Clinical measures included the Montgomery-Åsberg Depression Rating Scale (MADRS), Sheehan Disability Scale, and comorbid psychiatric diagnoses. SOC’s predictive value for relapse, suicide attempts, and self-harm was evaluated using data from a 7-year follow-up visit.

**Results:**

SOC scores were lower in individuals with bipolar disorder than in healthy controls (*p* < 0.001). Lower SOC correlated with greater functional impairment, residual depressive symptoms, and comorbid anxiety disorders. SOC was stable over 14 years, with modest increases in both groups. In the bipolar disorder group, baseline SOC predicted self-harm during the initial 7-year follow-up (*p* = 0.012) but was not associated with mood episode relapse or suicide attempts.

**Conclusion:**

Although individuals with bipolar disorder exhibit lower SOC than healthy controls, the difference probably reflects broader psychopathology rather than core bipolar symptoms. SOC predicted self-harm during the 7-year follow-up period, but not mood episode relapse or suicide attempts. Targeting SOC in interventions may benefit comorbid conditions, but is unlikely to impact bipolar-specific outcomes.

**Supplementary Information:**

The online version contains supplementary material available at 10.1186/s12888-025-06779-3.

## Introduction

The theoretical framework for the Sense of Coherence (SOC) rating scale was introduced by Antonovsky (1979). Individuals with a high SOC find the world predictable, comprehensible, meaningful, and manageable. This equips them psychologically to better draw upon internal or external resources to cope during times of difficulty [1]. Antonovsky suggested that such individuals are more resilient to stress and hardships than individuals with a low SOC. Since its introduction, SOC has been studied in relation to both somatic and psychiatric disorders. Previous research has shown a correlation between low SOC and various mental and somatic health conditions [2]. Conversely, a high SOC appears to be positively correlated with the quality of life in individuals with various illnesses and disabilities [3].

Regarding the predictive health-protective power of SOC, however, empirical evidence remains ambiguous. A Finnish study of young boys reported that lower SOC predicted subsequent anxiety, depression, antisocial personality, and substance use disorders [4]. Moreover, Edbom and colleagues [5] found that a high SOC predicted less ADHD symptoms in adolescents 5 years later. However, Kivimäki et al. [6] did not find support for longitudinal relationships between SOC and health in two groups of municipal and private sector employees. This raises the question whether SOC is a causal factor promoting health or merely co-varies with health. Clarifying this distinction is crucial because if high SOC causes better health, interventions aimed at increasing SOC could be an effective strategy for improving health outcomes.

Bipolar disorder is characterized by recurring mood episodes that significantly impair quality of life [7]. Further, individuals with bipolar disorder experience more somatic disease, such as cardiovascular diseases, and have higher rates of suicide and suicide attempts [8]. Research on SOC in bipolar disorder is limited, and it remains unclear whether SOC scores predict outcome or symptom severity in this population. Further, no study has compared SOC in individuals with bipolar disorder with that of a healthy control group.

The aims of our study were threefold. First, we aimed to compare SOC scores between mood-stable patients with bipolar disorder and healthy controls. Additionally, we sought to investigate the relationship between SOC score and clinical as well as demographic characteristics within the patient group. Second, we aimed to determine if SOC could predict episode relapse, suicide attempts, and self-harm in individuals with bipolar disorder over a 7-year follow-up period. Third, we assessed the stability of SOC scores over time in both individuals with bipolar disorder and healthy controls over a 14-year follow-up period.

## Methods

### Sample

Our study is part of the St. Göran bipolar project (SBP). A total of 113 controls and 248 patients with bipolar disorder from the SBP were included. Inclusion criteria were complete SOC questionnaires and, for the patient group, information on bipolar disorder subdiagnosis. Data were extracted from the St. Göran bipolar project database in March 2024.

The SBP recruited study participants from a specialized bipolar outpatient tertiary clinic at the Northern Stockholm psychiatric clinic, Stockholm, Sweden. The catchment area of this clinic encompasses a diverse range of backgrounds, from affluent regions to areas characterized by higher deprivation indices. All patients with suspected bipolar disorder initially receiving care at the Northern Stockholm psychiatric clinic were subsequently referred to this tertiary bipolar out-patient unit for work-up and further treatment. Both newly referred outpatients and continuing patients at the tertiary care bipolar out-patient unit diagnosed with bipolar disorder were invited to participate in the study.

A population-based comparison group was recruited by Statistics Sweden (SCB). Individuals in the comparison group were matched to patients by nationality (born in Sweden), hometown (living in the region of Stockholm), sex, and age. SCB was provided the national personal identification numbers for 139 patients and randomly selected seven possible control persons to each patient. SCB then mailed information about the study to the selected individuals. Those interested in participating in the study were asked to contact the research nurse, who conducted a screening telephone interview and scheduled a visit for a comprehensive evaluation.

The diagnostic procedures, clinical work-up, and study protocol have been described in detail previously [9]. Here, we focus on the methods and sources of information used in the analyses of the present study. All patients were in a stable mood during the study visits. Residual depressive and manic symptoms were assessed using the Montgomery-Åsberg Depression Rating Scale (MADRS) and Young Ziegler Mania Rating Scale (YMRS), respectively.

Information on diagnosis, symptom history, and co-morbidity were collected from the patient history form used in the SBP. Information on pharmacological treatment, height, and weight were also collected during study visits.

For a subset of patients, the SBP database holds information on episode relapse, suicide attempts, and self-harm collected at two follow-up visits, approximately 7 and 14 years after the baseline visit. Further, we used information on SOC, MADRS, YMRS, Global Assessment of Functioning Scale (GAF), and Sheehan disability scale collected both at the baseline visit and during the 14-year follow-up visit.

We used the 29-item version of the SOC scale, developed by Antonovsky [1], to measure SOC. Responses were reported on a 7-point Likert-type scale. Although the scale theoretically comprises three components — comprehension, manageability, and meaningfulness — subsequent research has shown that total SOC score cannot be reliably broken down into independent measures of these components [10]. We therefore used total SOC scores in our analyses and disregarded sub-scores of the scale. Further, although some studies have categorized SOC scores using data driven or theory-based cut-offs [10], consensus regarding clinically meaningful or reproducible categories remains elusive. Consequently, we restricted our analysis to continuous SOC scores. SOC scores were collected at the baseline and 14-year follow-up visits but were not available for the 7-year follow-up visit.

The Sheehan disability scale is a self-assessment tool [11] that measures the extent to which an individual’s mental health symptoms impair their work/school performance, social life, and family life/home responsibilities. It consists of three self-rated items, each scored on a 10-point visual analogue scale, where 0 indicates no impairment and 10 indicates extreme impairment.

Global Assessment of Function (GAF) [12] is an interviewer-rated visual scale ranging from 0 to 100. ‘0’ indicates insufficient information, ‘1’ indicates the lowest possible functioning, and ‘100’ represents the highest level of functioning. The scale considers both the patient’s psychopathology and social functioning.

### Statistical analyses

All statistical analyses were carried out using SPSS version 29.0.0.0. Group comparisons were performed using unpaired or paired t-tests, chi-square tests or Mann-Witney U-tests as appropriate. Single associations between demographic and clinical descriptors and SOC score were analyzed using Pearson correlations. Multivariate analysis of clinical descriptors and SOC score was performed using backward step-wise linear regression modeling.

## Results

### SOC in individuals with bipolar disorder and controls

The age and sex distribution were similar in the patient and control groups. Patients with bipolar disorder had significantly lower SOC scores compared to the control group (Table 1). SOC scores were associated with age (*R* = 0.11, *P* = 0.04) but not with sex (*R*=-0.09, *P* = 0.10) in the total sample.

**Table 1:** SOC in individuals with bipolar disorder and controls

	Controls (*N* = 113)	Patients (*N* = 248)	t	χ2	*P*-value
SOC (*N*, %)	158 (18)	130 (28)	-11.4^1^		2.8*10^− 25^
Female (*N*, %)	62 (55)	153 (62)		1.5	0.22
Age (mean, SD)	38 (13)	38 (13)	0.25		0.80

^1^Equal variances not assumed

### SOC and clinical and demographic characteristics

Within the patient group, the SOC score was lower in patients with a bipolar type 1 subdiagnosis and in those with history of suicide attempt. Further, SOC score correlated negatively with MADRS, GAF (both function and symptom scores), and Sheehan disability scale scores. Finally, SOC scores were lower in those with stimulant or benzodiazepine treatment, and in those with comorbid conditions, including current or past social phobia, panic disorder, obsessive-compulsive disorder (OCD), generalized anxiety disorder (GAD), post-traumatic stress disorder (PTSD), or attention-deficit/hyperactivity disorder (ADHD) (see Table 2).

**Table 2:** Associations between SOC and clinical characteristics of the bipolar disorder group

Dependent variable	*N* (%)	Statistics	
		Pearson *r*	*P*-value
BP1 subdiagnosis	134 (37)	-0.18	0.001
History of self-harm with or without suicide intent^1^	88 (24)	-0.24	2.2*10^− 4^
Somatic comorbidity^2^	66 (18)	-0.06	0.41
University education^3^	130 (36)	-0.007	0.91
Employed^4^	146 (40)	0.08	0.26
	**Mean (SD)**		
BMI^5^	25 (4)	-0.03	0.67
GAF_F_	67 (11)	0.36	3.0*10^− 8^
GAF_S_	67 (10)	0.32	6.3*10^− 7^
MADRS^5^	7 (7)	-0.46	7*10^− 13^
YMRS^5^	1 (2)	-0.13	0.056
Sheehan disability score^5^	13 (9)	-0.59	1.9*10^− 22^
Life-time depressive episodes^5^	10 (15)	-0.12	0.069
Life-time manic episodes^5^	2 (3)	0.06	0.34
Life-time hypomanic episodes^5^	7 (13)	-0.10	0.13
**Pharmacological treatment**	***N*** **(%)**		
Lithium	140 (56)	0.1	0.12
Antiepileptic	80 (32)	-0.10	0.10
Antipsychotic	59 (24)	-0.09	0.15
Antidepressant	98 (40)	-0.08	0.19
Stimulant	7 (3)	-0.18	0.004
Bensodiazepine	36 (15)	-0.19	0.003
**Current or past co-morbidity**	***N*** **(%)**		
Panic disorder^4^	63 (28)	-0.24	2.3*10^− 4^
Social phobia^4^	38 (17)	-0.24	2.2*10^− 4^
OCD^7^	24 (11)	-0.20	0.002
GAD^2^	34 (15)	-0.36	3.7*10^− 8^
PTSD^4^	8 (4)	-0.15	0.021
Autism^8^	5 (2)	-0.10	0.13
ADHD^9^	29 (13)	-0.22	0.001
Borderline personality disorder^6^	4 (2)	0.05	0.49
Other personality disorders^4^	12 (5)	-0.11	0.10

Missing values for ^1^19 individuals, ^2^23 individuals, ^3^2 individuals, ^4^21 individuals, ^5^20 individuals, ^6^10 individuals, ^7^22 individuals, ^8^24 individuals, and ^9^25 individuals

All descriptors with a correlation *p*-value < 0.25 were entered into a backward step-wise linear regression model with SOC score as the dependent variable. Age was added as a co-variate as it was associated with SOC score in the overall study population. The final model (F = 37.3, *p* = 1.1*10^− 22^) explained 44% of the variation in SOC scores and included MADRS (beta = -0.17, *p* = 0.008), Sheehan disability scale (beta = -0.50, *p* = 1.6*10^− 12^), GAD (beta = -0.15, *p* = 0.012), and age (beta = 0.15, *p* = 0.007) as statistically significant regression coefficients.

### Outcome prediction in individuals with bipolar disorder

Lower baseline SOC scores were significantly associated with the incidence of self-harm (*p* = 0.012) over the initial 7-year follow-up period (Table 3). However, neither suicide attempts (*p* = 0.18) nor depressive (*p* = 0.27), hypomania (*p* = 0.11), mania (*p* = 0.1) episode relapse, or in-patient care during follow-up were associated with baseline SOC scores.

**Table 3:** Correlation between baseline SOC and events during the first 7-year follow up period

Event	Study participants (*N* = 131) with at least one event, *N* (%)	Correlation with baseline SOC score	
		R	*P*-value
Suicide attempt	11 (8)	-0.12	0.18
Self-harm^1^	21 (16)	-0.22	0.012
Depressive episode^2^	86 (69)	-0.1	0.26
Hypomanic episode^3^	40 (34)	-0.15	0.11
Manic episode^4^	32 (27)	0.15	0.1
In-patient care	49 (37)	0.1	0.28

Missing values for ^1^2 individuals, ^2^6 individuals, ^3^13 individuals, and ^4^14 individuals.

### SOC at the 14-year follow-up visit

Table 4 shows that SOC scores at the 14-year follow-up visit correlated significantly with baseline SOC scores in both the patient and control groups. SOC scores were higher at the follow-up visit compared to baseline in both groups. In the patient group, Sheehan disability scale scores also correlated between visits and had similarly increased at follow-up. MADRS, YMRS, and GAF were not statistically different between visits and only GAF correlated weakly between visits.

**Table 4:** Correlation between ratings at baseline and 14-year follow up visit

**Patient group**						
	**Baseline mean (SD)**	**14-year follow-up mean (SD)**	**Pearson correlation**		**Paired t-test**	
			**R**	***P*** **-value**	**T**	***P*** **-value**
SOC	127 (27)	140 (23)	0.63	2.6*10^− 8^	4.7	1.3*10^− 5^
Sheehan disability score	14 (9)^1^	9 (8)^1^	0.49	5.6*10^− 5^	-4.6	2.6*10^− 5^
MADRS	6 (7)^2^	5 (5)	0.14	0.30	-1.0	0.34
YMRS	1 (2)^3^	0 (1)	0.22	0.10	-1.9	0.06
GAF_F_	67 (10)^2^	66 (15)^4^	0.30	0.021	-0.4	0.66
**Control group**						
	**Baseline mean (SD)**	**14-year follow-up mean (SD)**	**Pearson correlation**		**Paired t-test**	
			**R**	***P*** **-value**	**T**	***P*** **-value**
SOC	158 (19)	165 (17)	0.62	2.3*10^− 7^	3.3	0.002

Only individuals with SOC ratings for both baseline and 14-year follow up visits are included in the table (patient group *n* = 63 and control group *n* = 57). Missing data for ^1^one individual, ^2^three individuals, ^3^four individuals, and ^4^two individuals

### Dropout analysis

All patients included at baseline were intended for inclusion in follow-up assessments, but there was a gradual loss to follow-up over the 7- and 14-year visits. This potentially non-random attrition may introduce bias in comparisons between baseline and follow-up. To address this, we compared the clinical characteristics at baseline between patients who completed the 14-year follow-up visit and those who did not. As shown in supplemental Table 1, however, there were few statistically significant differences between completers and drop-outs in terms of baseline clinical characteristics.

## Discussion

We assessed SOC over 14 years in individuals with bipolar disorder and healthy controls. The main findings were: First, individuals with bipolar disorder scored lower on the SOC scale compared to the control group. Second, baseline SOC scores correlated strongly with SOC scores at the 14-year follow-up visit in both groups. Third, baseline SOC scores were negatively associated with self-harm during the first 7 years of follow up among patients with bipolar disorder. Finally, within the bipolar disorder group, lower baseline SOC scores were associated with greater self-rated functional impairment, more residual depressive symptoms, and comorbid anxiety disorders, while age was positively associated with higher baseline SOC scores.

No previous studies have directly compared SOC scores between individuals with bipolar disorder and healthy controls. Nonetheless, the lower SOC scores observed in the bipolar disorder group align with previous studies that have linked low SOC scores to an increased risk of developing a psychiatric disorder [4, 13]. However, the association between SOC and psychopathology remains a topic of debate. While one previous study suggested that SOC score remains independent of conditions like depression and anxiety [14], supporting its predictive qualities, other studies have demonstrated associations between lower SOC scores and various forms of psychopathology [15, 16]. We found no associations between SOC score and the number of previous mood episodes. However, we did find associations between SOC score and baseline residual depressive symptoms, as well as anxiety disorder comorbidity. These findings suggest a general association between low SOC and psychopathology, though not specifically with the core symptoms of bipolar disorder.

Regarding the potential predictive value of SOC, we found no correlations between SOC scores and mood episode relapses or suicide attempts during the follow-up period. Again, this suggests that a high SOC may not necessarily reduce the risk for episode relapse or suicide attempts in individuals with bipolar disorder.

Nevertheless, we did identify a negative correlation between SOC scores and instances of self-harm, suggesting some predictive validity of SOC in our study. Previous work has found that low SOC can predict future psychiatric disorder diagnoses in previously undiagnosed individuals [13]. Additionally, while low SOC is recognized as a risk factor for negative health outcomes, studies suggest that high SOC may not confer additional benefits over an average SOC [17, 18].

Longitudinal studies indicate that SOC is a relatively stable trait [19]. However, exposure to traumatic events later in life may decrease SOC [20] and a recent study suggests that exercise training can increase SOC in individuals with bipolar disorder [21]. Previous work has also shown a general age-related increase in SOC [22]. In our study, we observed an increase in SOC over time in both individuals with bipolar disorder and healthy controls. Further, baseline age showed a weak but positive association with SOC in the total study population and remained a significant predictor for SOC in the patient group, even after accounting for clinical variables. However, it is important to consider that selective drop-out between our follow-up visits could influence these findings: It cannot be ruled out that individuals who remained in the study might have been more likely to experience an increase in SOC scores.

If SOC represents a causal salutogenic factor, interventions aimed at increasing SOC could potentially improve outcomes in psychiatric disorders. Previous work suggests a general association between low SOC and psychiatric disorders [6, 13]. Similarly, we found an association between low SOC and comorbid psychiatric conditions in the patient group, but we found no association between SOC and the core symptoms of bipolar disorder. However, associations between more domain specific symptoms and SOC have been reported. For example, low SOC has been associated with a high level of basic self-disturbances [23] or borderline personality disorder traits [24]. The latter finding is consistent with our observed increased risk of self-harm. Thus, improving SOC in individuals with bipolar disorder could be beneficial for certain comorbid psychiatric conditions. Further research is needed to determine which other aspects of psychiatric psychopathology are associated with SOC, and to explore how SOC relates to outcomes such as quality of life in individuals with psychiatric disorders.

We used data from the St. Göran study, where both patient and control groups were meticulously phenotyped, and information was collected according to a standardized study protocol. A key strength of this study is the inclusion of longitudinal data, allowing us to assess the stability and predictive power of SOC scores. However, the lack of SOC data at the 7-year follow-up, and the drop-out rate are limitations. Drop-outs during a 14-year study period are inevitable, but our comparison of baseline characteristics showed no major differences between those who remained in the study and those who dropped out. Nevertheless, drop-out could be non-random and introduce bias. Additionally, the study’s outcome measures were not salutogenic, limiting our ability to evaluate the health promoting effects of high SOC [10]. Finally, the combined assessment of self-harm and suicide attempts at baseline complicates the interpretation of their association with SOC.

## Conclusions

In conclusion, our findings indicate that individuals with bipolar disorder have a lower SOC compared to a control group. SOC was not associated with core symptoms of bipolar disorder or the risk of episode relapse during a 14-year follow-up. However, SOC was associated with residual depressive symptoms, comorbid anxiety disorder, functional impairment, and predicted self-harm during the follow-up. Thus, SOC seems to be linked to general psychopathology, and interventions targeting SOC are unlikely to impact mood episodes or suicide risk in individuals with bipolar disorder.

## Electronic supplementary material

Below is the link to the electronic supplementary material.

Supplementary file 1: Table 1 Baseline clinical characteristics in individuals with bipolar disorder who completed the 14-year follow-up visit compared to individuals who did not complete the 14-year follow-up visit

## Data Availability

Access and use of research data is regulated by specific national legislation in addition to general data protection legislation. Research data in the SBP is controlled by the University of Gothenburg, which handles questions on access to and use of data. For researchers, please contact the corresponding author for more information on data sharing and access to meta-data for the SBP.

## Abbreviations

ADHD: Attention–deficit/hyperactivity disorder
GAD: Generalized anxiety disorder
GAF: Global Assessment of Functioning
MADRS: Montgomery–Åsberg Depression Rating Scale
OCD: Obsessive–compulsive disorder
PTSD: Post–traumatic stress disorder
SBP: St. Göran bipolar project
SCB: Statistics Sweden
SOC: Sense of coherence
YMRS: Young Ziegler Mania Rating Scale
