# Supplementary material for: Sense of coherence in bipolar disorder– a longitudinal study
Source: BMC Psychiatry. 2025 Apr 2;25:324. doi: 10.1186/s12888-025-06779-3 (PMC11966924; doi:10.1186/s12888-025-06779-3)
Supplement: Supplementary file 1 — Table 1 Baseline clinical characteristics in individuals with bipolar disorder who completed the 14-year follow-up visit compared to individuals who did not complete the 14-year follow-up visit [file 12888_2025_6779_MOESM1_ESM.docx]

**Supplementary table 1. Baseline clinical characteristics in individuals with bipolar disorder who completed the 14-year follow-up visit compared to individuals who did not complete the 14-year follow-up visit**

|  | Follow-up (N=63) | Drop-out (N=185) | **Statistics** | |
| --- | --- | --- | --- | --- |
|  | **Mean (SD)** | | **t** | **P** |
| SOC | 127 (27) | 130 (28) | -0.9 | 0.36 |
| BMI^1^ | 26 (4) | 25 (4) | 1.6 | 0.11 |
| GAF_F_^2^ | 67 (10) | 67 (11) | -0.09 | 0.93 |
| GAF_S_^2^ | 67 (10) | 67 (10) | 0.7 | 0.46 |
|  |  |  | **U** | **P** |
| MADRS^1^ | 6 (7) | 7 (8) | 4886 | 0.94 |
| YMRS^1^ | 1 (2) | 1 (2) | 4581 | 0.64 |
| Sheehan disability score^1^ | 13 (9) | 13 (9) | 5314 | 0.45 |
| Life-time depressive episodes^1^ | 13 (19) | 9 (13) | 5375 | 0.49 |
| Life-time manic episodes^1^ | 2 (3) | 2 (3) | 5114 | 0.97 |
| Life-time hypomanic episodes^1^ | 8 (14) | 6 (12) | 5412 | 0.47 |
| EtOH consumption^1^ | 3 (7) | 4 (9) | 5023 | 0.91 |
|  | **N (%)** | | **χ^2^** | **P** |
| BP1 subdiagnosis | 33 (52) | 101 (55) | 0.09 | 0.76 |
| History of suicide attempt or self-harm^3^ | 18 (30) | 70 (41) | 2.4 | 0.12 |
| Somatic comorbidity^4^ | 17 (28) | 49 (30) | 0.04 | 0.84 |
| University education^5^ | 37 (59) | 93 (50) | 0.87 | 0.35 |
| Employed^6^ | 40 (68) | 106 (63) | 0.42 | 0.52 |
| **Pharmacological treatment** | **N (%)** | | χ^2^ | P |
| Lithium | 38 (60) | 102 (55) | 0.51 | 0.47 |
| Antiepileptic | 20 (32) | 60 (32) | 0.01 | 0.92 |
| Antipsychotic | 15 (24) | 44 (24) | 0 | 1.0 |
| Antidepressant | 23 (37) | 75 (42) | 0.32 | 0.57 |
| Stimulant | 2 (3) | 5 (3) | 0.04 | 0.85 |
| Bensodiazepine | 8 (13) | 28 (15) | 0.23 | 0.64 |
| **Co-morbidity** | **N (%)** | | χ^2^ | P |
| Panic disorder^6^ | 20 (33) | 43 (32) | 0.02 | 0.89 |
| Social phobia^6^ | 9 (15) | 29 (17) | 0.18 | 0.67 |
| OCD^7^ | 7 (12) | 17 (10) | 0.09 | 0.76 |
| GAD^2^ | 9 (15) | 25 (15) | 0.001 | 0.97 |
| PTSD^6^ | 2 (3) | 6 (4) | 0.009 | 0.93 |
| Autism^8^ | 0 (0) | 5 (3) | 1.8 | 0.18 |
| ADHD^9^ | 8 (14) | 21 (13) | 0.04 | 0.84 |
| Personality disorder^3^ | 1 (2) | 11 (7) | 2.1 | 0.14 |

Missing values for ^1^20 individuals ^2^18 individuals, ^3^19 individuals, ^4^23 individuals, ^5^2 individuals, ^6^21 individuals, ^7^22 individuals, ^8^24 individuals, and ^9^25 individuals.
